# Advanced breast cancer

**DOI:** 10.11604/pamj.2018.29.226.14239

**Published:** 2018-04-25

**Authors:** Nora Naqos

**Affiliations:** 1 Department of Medical Oncology, Mohammed VI University Hospital, Marrakech, Maroc

**Keywords:** Breast, advanced, cancer

## Image in medicine

Breast cancer is the most common type of cancer in women worldwide and its advanced stage is the first cause of cancer death in women. We report a case of a 65-year-old woman who presented nodular lesions in the right breast 8 months ago, progressively increasing in size and number, a biopsy shows the presence of breast cancer that was an invasive ductal carcinoma SBR 2 triple negative. CT scan showed lung and liver metastasis. She underwent chemotherapy based on anthracycline with cyclophosphamide, we observed a progression of the skin lesions after 3 cycles. A chemotherapy based on taxane was started. Breast cancer is the first cancer in woman; its bad evolution should lead to an effective screening of lesions in an early stage so as to improve both survival and quality of life.

**Figure 1:**
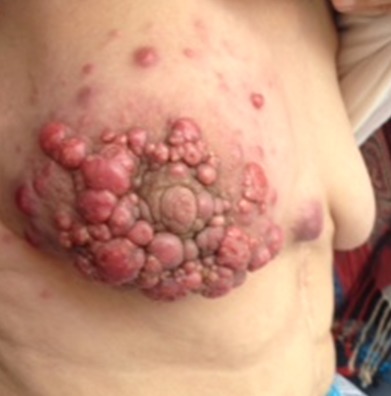
Nodular lesions in the right breast

